# Markerless Motion Capture Parameters Associated with Fall Risk or Frailty: A Scoping Review

**DOI:** 10.3390/s25185741

**Published:** 2025-09-15

**Authors:** Emma Osness, Serena Isley, Jennifer Bertrand, Liz Dennett, Jack Bates, Nathan Van Decker, Alexis Stanhope, Ayushi Omkar, Naomi Dolgoy, Victor E. Ezeugwu, Puneeta Tandon

**Affiliations:** 1 Liver Unit, Division of Gastroenterology, Faculty of Medicine and Dentistry, University of Alberta, Edmonton, AB T6G 2R3, Canada; eosness@ualberta.ca (E.O.); isley@ualberta.ca (S.I.); jbertran@ualberta.ca (J.B.); jbates1@ualberta.ca (J.B.); ayushi@ualberta.ca (A.O.); 2 Geoffrey and Robyn Sperber Health Sciences Library, University of Alberta, Edmonton, AB T6G 2R3, Canada; liz.dennett@ualberta.ca; 3 Faculty of Medicine and Dentistry, University of Alberta, Edmonton, AB T6G 2R3, Canada; nvandeck@ualberta.ca (N.V.D.);; 4 Faculty of Rehabilitation Medicine, Department of Occupational Therapy, University of Alberta, Edmonton, AB T6G 2R3, Canada; dolgoy@ualberta.ca; 5 Faculty of Rehabilitation Medicine, Department of Physical Therapy, University of Alberta, Edmonton, AB T6G 2R3, Canada

**Keywords:** fall risk, frailty, digital health, markerless motion capture, kinematics

## Abstract

Frailty (a syndrome resulting in reduced physical function) assessments and fall risk assessments rely heavily on in-person evaluations and subjective interpretation, limiting scalability and access. Markerless motion capture (MMC) offers a promising solution for remote, objective assessment, but key kinematic parameters associated with frailty and fall risk remain unclear. This scoping review synthesized evidence from MEDLINE, Embase, Scopus, and CINAHL (inception to October 2024). Eligible studies used MMC to assess adults and compared outcomes to validated frailty or fall risk measures. Of 8048 studies, 39 met the inclusion criteria: 30 evaluated fall risk, 7 evaluated frailty, and 2 evaluated both, including 3114 participants (mean age 75.8; 42% male). Microsoft Kinect was used in 75% of the studies. An average of 23 features was extracted per study. Gait analysis was the most common MMC assessment for fall risk, identifying gait speed, stride length, and step width as key parameters. Frailty-related features were less consistent, with two studies identifying power, speed degradation, power reduction, range of motion, and elbow flexion time during a 20 s arm test. Future studies require standardization of methods and improved reporting of data loss. Despite the emerging nature of the field, MMC shows potential for the identification of fall risk and frailty.

## 1. Introduction

Frailty is a clinical syndrome characterized by reduced physical function and impaired health that increases in prevalence as individuals age [1,2]. It is associated with increased morbidity and mortality [3,4], hospitalization, and financial burden on healthcare systems [5,6]. Falls, closely linked to aging, affect approximately one-third of older adults annually [7], with fall risk influenced by many factors [8]. Frailty and fall risk frequently co-occur and are two of the most common geriatric syndromes among community-dwelling adults [7,9]. As the global population ages, both frailty and fall risk are growing public health concerns. In Canada, fall rates among adults aged 65 years and older have risen, with a 111% increase in fall-related mortality between 2001 and 2019 [10]. Frail individuals are at an increased risk of falling compared to their non-frail peers [11,12]. Furthermore, frail individuals have a higher likelihood of serious injury, fracture, and mortality following a fall [13,14]. Both frailty and falls influence each other [15] in that falls can precipitate a “fear of falling” that leads to decreased physical activity, fueling further muscle loss and advancing frailty, and, in turn, a greater risk of falls [16]. Evidence suggests that by examining these conditions together, there is more accurate risk stratification, facilitating highly targeted interventions that optimize patient outcomes [16,17]. Indeed, interventions in individuals at risk of frailty and falls have been shown to reduce healthcare burden and improve quality of life [18,19,20].

Frailty and fall risk are most commonly assessed using a range of self-report or performance-based approaches [21]. The absence of a single, standardized reference method has contributed to substantial variability in how these conditions are measured [21,22]. Given that impairments in physical function underpin both frailty and fall risk [23,24], this creates an opportunity to improve evaluation through objective, sensitive, and standardized assessment tools grounded in physical function [21,25].

Technologies capable of objectively capturing movement offer a promising approach forward. While marker-based motion capture remains the “gold standard” of optical motion tracking technology, it is limited by the need for specialized equipment (e.g., multiple infrared cameras) at high costs, time-intensive setups with large physical space requirements, and mobility limitations during testing [26,27]. Inertial measurement units (IMUs), which collect data from an accelerometry sensor (or sensors) placed on the body, also provide a valuable movement signal. However, they can be susceptible to drift and signal noise, which can affect measurement accuracy in some settings [28].

Markerless motion capture (MMC) has emerged as a scalable and accessible alternative. By using simple cameras in conjunction with pose estimation algorithms, MMC eliminates the need for physical markers or sensors. Its compatibility with smartphones and other devices [29] enhances its feasibility for clinical and in-home settings [30]. One of the most widely used MMC tools is the Kinect [31], a consumer-grade motion sensing device that was originally developed for video game applications [32]. The Kinect integrates an RGB camera, a depth sensor, and sound capture, allowing simultaneous collection of multimodal data. This versatility has supported its adoption in both research and clinical settings [33].

Evaluating whether MMC can achieve accuracy comparable to established motion capture methods has been a central focus of validation studies. A recent meta-analysis of 22 studies found that MMC demonstrates excellent inter-rater reliability for key spatiotemporal gait parameters (intraclass correlation coefficient (ICC) = 0.81–0.99) and excellent concurrent validity (ICC = 0.98) when compared with marker-based motion capture [34]. Similarly, a systematic review of 20 studies reported high agreement between MMC and marker-based systems, particularly for spatiotemporal outcomes, further reinforcing the robustness of these findings [35]. IMUs can provide high precision for specific metrics, such as lower limb joint angles, but they are less reliable for capturing complex movements or multiplane joint rotations—an area where MMC performs particularly well [36]. Balancing accessibility and robust accuracy, MMC represents a practical tool for clinical application.

Moreover, MMC enables the extraction of kinematic features—quantitative descriptors of movement (e.g., speed, step length, joint angles, or timing parameters) derived from temporospatial data. These features act as digital biomarkers, translating raw motion capture into objective and clinically interpretable indicators of physical function [37]. They have demonstrated relevance across a range of health conditions, including frailty and fall risk [38]. For example, shorter step length and slower speed-to-sit times distinguish individuals at higher versus lower fall risk [39,40], while other features have improved diagnostic accuracy in musculoskeletal disorders and post-stroke impairments [41,42,43]. Beyond specific conditions, kinematic features enhance standardization in movement assessment, which is imperative when evaluating frailty and fall risk [44,45]. Collectively, this growing evidence positions kinematic features as reliable indicators of patient health and functional status [46,47], underscoring their importance for systematic investigation.

Despite the growing recognition of kinematic features as meaningful digital biomarkers, there has been limited synthesis of the specific features that are most relevant to frailty and fall risk. Existing reviews have focused on broader healthcare applications of MMC [30], on particular clinical conditions (e.g., neurodegenerative disease) [48], or on alternative technologies, such as IMUs [49]. To address this gap, our primary aim was to summarize the kinematic features measured through MMC that are associated with fall risk and frailty. In addition, because the utility of these features depends on more than their identification alone, we examined several practical and methodological considerations specific to extracting kinematic features in this context. We explored how MMC assessments were set up across studies to determine whether standardized approaches are emerging or whether practices remain highly variable. We also examined whether findings were reported in a stratified manner (e.g., by age, sex, body mass index, or disease status), which allows for understanding whether MMC-derived features are generalizable beyond narrow samples. Finally, we reviewed reported rates and reasons for incomplete or poor-quality MMC recordings, since high levels of unusable data could limit feasibility in clinical practice. Together, these secondary aims provide context on whether MMC-derived kinematic features of frailty and fall risk are not only valid but also practical, generalizable, and feasible for broader clinical use. A preliminary search of MEDLINE, Prospero, Google Scholar, and Open Science Framework found no existing or ongoing systematic or scoping reviews on this topic. Given the breadth of our objectives, a scoping review approach was selected.

## 2. Methods

### 2.1. Study Design

This scoping review was conducted in accordance with the *JBI manual for evidence synthesis* [50]. The research question was developed using the PEOs (population, exposure, outcome, study design) framework. This review followed the Preferred Reporting Items for Systematic Reviews and Meta-Analysis Extension for Scoping Reviews (PRISMA-SCR) guidelines [51].

### 2.2. Primary Research Question

What markerless motion capture kinematic features are associated with frailty and/or fall risk in adults?

### 2.3. Secondary Research Questions

1. How was the MMC technology set up for assessments (e.g., camera type, angle, distance from participant)?
2. Were the results stratified by demographic or disease characteristics (sex, age, body mass index, etc.) and, if so, what differences were reported?
3. What were the reported rates and reasons (if any) for poor or incomplete MMC data recording, and what reasons were provided (if any)?

### 2.4. Search Strategy

A search strategy was developed with guidance from a health sciences librarian, LD, alongside clinical input from authors PT and VE. Text words from titles and abstracts and relevant index terms were used to construct the initial MEDLINE search strategy, which was then adapted for the other databases. Four databases (MEDLINE, CINAHL, Embase, and Scopus) were searched with no date restrictions from inception to October 2024. The reference lists of all the included studies were also screened. All search results were imported into Covidence systematic review management software (Veritas Health Innovation, Melbourne, Australia) for screening and data management. A copy of the search strategy can be found in the Supplementary Materials.

### 2.5. Eligibility Criteria

#### The Inclusion Criteria Consisted of the Following

Population: Adults (≥18 years) of any health status.

Exposure: Movement assessment using MMC (all forms of MMC assessment included).

Outcome: A fall risk or frailty reference tool with published evidence of reliability and validity.

Study Design: Original research articles.

### 2.6. Exclusion Criteria

Studies involving animals, pediatric populations, or cadaveric models were excluded.

Studies using only marker-based motion capture, IMUs, force plates, or radar systems were excluded.

Literature reviews, conference abstracts without an associated full paper, or non-English publications were excluded.

### 2.7. Study Selection

**Figure 1:**
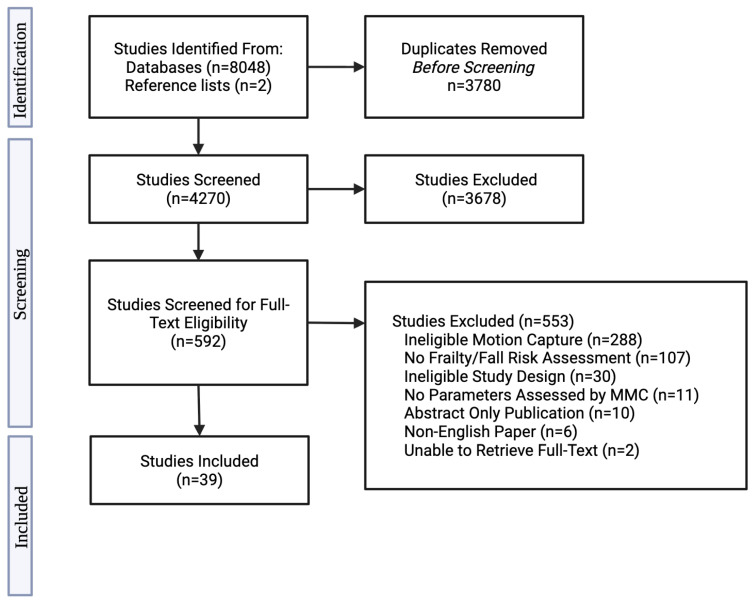
Flow diagram of this review. n: number.

Following the search, all identified citations were collated and uploaded to Covidence, where duplicate records were removed. In accordance with the JBI manual for evidence synthesis, a pilot screening of 25 papers was conducted to assess inter-reviewer agreement [50]. Once a 75% consensus was reached, title and abstract screening was started. Two independent reviewers (EO, SI) carried out title–abstract screening and full-text review. Reasons for the exclusion of articles at full text are recorded and summarized in Figure 1. Disagreements were resolved through discussion or by a third independent reviewer (JB, VE, PT). The results of the search and the study inclusion process are reported in full and presented in a PRISMA-ScR flow diagram (Figure 1) [51].

### 2.8. Data Extraction

An initial extraction trial ensured that all relevant data were captured. Following this, a single reviewer extracted data from all the included studies. To ensure consistency and validity, a second reviewer independently verified 20% of the extracted studies. The extraction tool was developed by the review team and included the following categories: study characteristics: author, country, year, study design, and relevant conclusions; participant characteristics: age, health status, BMI, sex, sample size, and fall risk and/or frailty status; technology characteristics: device used, number of devices, frame rate, resolution, extraction methods, key-points, features, and additional equipment; assessment characteristics: frailty or fall risk assessment used, administration details (location of testing, set-up requirements), associated movement task performed with MMC (any test involving motion capture with MMC was deemed eligible) and reported accuracy, specificity, and sensitivity; and MMC parameters identified (number of parameters significantly related to fall risk/frailty and number of parameters non-significantly related to fall risk/frailty). The significance or non-significance of parameters was extracted directly from the original papers (no additional statistical tests were performed as part of this review).

### 2.9. Definition of Terms

During the screening and extraction process, the following terms and definitions were used to guide the reviewers. *Clinician* was used to define any qualified practitioner who conducted assessments (e.g., nurse, certified exercise professional, physician). *Kinematic feature* was used to refer to any specific quantifiable aspect of movement (e.g., gait speed, joint angle). *Feature sets* were a collection of quantifiable kinematic features, as grouped by the original study authors (commonly used in papers about predictive modeling and machine learning). *Key points* referred to specific anatomical landmarks used to estimate human skeletal position during MMC [52]. A *validated reference method* was considered any fall risk or frailty assessment supported by empirical evidence of reliability and validity. Common reference methods of fall risk included the Timed Up and Go (TUG) [53], the Berg Balance Scale (BBS) [54], and fall history [55]. Common reference methods of frailty included Fried’s Frailty Phenotype (FFP) [56] and the Clinical Frailty Scale (CFS) [57]. We also included other methods of classification, provided they had reasonable evidence supporting their validity and reliability in assessing fall risk or frailty (e.g., the Frailty Meter [58]).

## 3. Results

The search across all four databases yielded 8050 results. After removing 3780 duplicates (including 15 identified manually), 4720 articles remained for title and abstract screening. Of these, 3682 were excluded, and 585 full-text articles were reviewed, with 37 meeting the inclusion criteria. An additional two studies were identified through reference list screening, resulting in 39 included studies. The study selection process is illustrated in a PRISMA-ScR flow diagram (Figure 1), including reasons for full-text exclusions.

### 3.1. Study and Population Characteristics

The 39 included studies were published between 2011 and 2024, across 17 countries, including the United States of America (n = 8) [58,59,60,61,62,63,64,65], Canada (n = 4) [66,67,68,69], Japan (n = 4) [70,71,72,73], Switzerland (n = 3) [39,40,74], China (n = 3) [75,76,77], Austria (n = 3) [78,79,80], Italy (n = 2) [81,82], Spain (n = 2) [83,84], Australia (n = 2) [85,86], and India (n = 2) [87,88], with one study each from Belgium [89], France [90], Brazil [91], South Korea [92], Malaysia [93], Taiwan [94], and Finland [95]. The study and population characteristics are summarized in Table 1. Across studies, a total of 3114 participants were included, with a mean age of 75.8 years (SD = 8.9); 42.6% of the participants were male. The sample sizes ranged from 6 to 437, with a mean of 79 (SD = 107). Among the participants, 565 (18.1%) were at risk of falls, and 550 (17.7%) were classified as frail. Most studies (n = 21) focused on older adults [40,59,60,61,62,63,64,67,73,75,77,78,79,80,82,88,89,90,92], while other studies included clinical populations, such as stroke (n = 4) [83,85,86,94], Parkinson’s disease (n = 3) [71,76,84], dementia (n = 3) [66,67,69], heart failure (n = 1), chronic obstructive pulmonary disease (n = 1) [58], and neuropsychological disorders (n = 1) [95]. Four studies did not specify specific health statuses of their population, describing them as hospital in-patients (n = 2) [39,74], bone clinic patients [65], and fallers/non-fallers [93]. One study included a younger population of university students [70]. Only 12 (31%) studies reported body mass index (BMI) [58,60,67,68,72,73,76,77,78,80,88,91,92], with an average of 25.0 kg/m^2^. One study specified the ethnicity of its participants, all of whom were Caucasian [63]. No studies reported stratified findings by demographic characteristics.

### 3.2. Technology Characteristics

The technology characteristics (addressing secondary research question one) of the studies are summarized in Table 2. Kinect was the most commonly used MMC device (n = 30; 75% of studies), including iterations such as the Microsoft, Azure, and Xbox 360 Kinect systems [39,40,59,61,62,63,64,65,66,68,69,73,75,76,77,78,79,80,81,82,83,84,85,86,88,89,90,94,95,96]. Other studies used different mobile devices and cameras [58,60,67,70,72]. One study did not specify device details [71]. Kinect-based skeletal tracking was the most frequently used extraction algorithm (n = 14) [59,63,65,66,68,73,75,77,78,79,80,85,89,90], followed closely by custom-made extraction algorithms (n = 13) [39,40,61,62,64,70,76,82,83,86,88,95,96]. OpenPose software and AlphaPose software (n = 4) were less commonly used [58,67,69,72]. One study used Google’s MediaPipe [60], and another used Unity3D software (Unity Technologies, San Francisco, CA, USA) [94]. Two studies did not report their pose estimation software [69,81]. Before 2020, Kinect accounted for 96% of the devices used; after 2020, other technologies became more common, and Kinect devices only comprised 58% of the usage. The set-up between studies varied widely, including sagittal plane motion capture (n = 5) [40,58,60,90,96] and frontal plane motion capture (n = 12) [65,77,78,79,80,81,84,86,88,89,92,95] (the remainder of the studies did not specify the plane of motion of MMC).

### 3.3. Assessment Characteristics

The assessment characteristics of the studies are summarized in Table 3. Most studies (n = 12) [59,69,72,76,81,84,85,89,90,94,96] used clinician-administered reference assessments for fall risk or frailty, while five studies did not specify the assessor [58,70,75,93,95]. The Timed Up and Go (TUG) test (n = 16) was the most frequently used validated reference measure for assessing fall risk [62,63,64,67,75,82,85,86,89,90,94,96]. Gait analysis was the most commonly employed MMC task or activity across all studies (n = 13) [61,62,64,66,67,68,71,72,77,83,84,85,91]. Most studies (n = 32) used clinical reference measures to assess frailty or fall risk [58,59,60,62,63,64,67,70,72,75,76,78,80,81,84,85,86,89,90,93,94,95,96], while the remainder (n = 7) relied on self-report reference measures [61,69,71,77,79,82,92].

### 3.4. Accuracy, Sensitivity, and Specificity

There was little consistency in the reporting of accuracy, sensitivity, and specificity of MMC assessment. Only seven studies reported accuracy values [39,70,72,77,81,92,95], which ranged from 70 to 84%, with an average of 76% (standard deviation: 6.4%). Five studies reported specificity (range 50–83%, mean 71% (SD: 13%)) [73,76,77,92,95]. Five studies reported sensitivity (range 45–86%, mean 72% (SD: 16%)) [73,76,77,92,95].

### 3.5. Statistical Analyses Reported

Statistical analysis varied widely across the studies. Most commonly, the studies used forms of correlational analysis (n = 24) to compare kinematic features to fall risk or frailty measures. The correlational statistics included Pearson’s correlations (n = 11) [58,62,66,67,73,78,80,82,83,88,89,97], Spearman’s correlations (n = 5) [65,71,73,86,89], and intraclass correlation coefficients (n = 5) [60,63,72,86,90]. Two studies did not specify the form of statistical correlation used; rather, the authors stated they used a form of “correlation” [64,93]. Other studies (n = 23) used group comparison tests like the Student’s *t*-test (n = 10) [39,40,71,75,76,79,83,84,92,96], analysis of variance (ANOVA) (n = 4) [73,80,84,91], multivariate ANOVA (n = 2) [40,96], analysis of covariance (n = 1) [78], the Wilcoxon rank sum test (n = 3) [39,40,96], and the Mann–Whitney U test (n = 4) [65,71,75,76].

Regression or association statistical models were used 12 times across the included studies. This included Poisson regression (n = 2) [66,69], hazard ratios (n = 1) [68], odds ratios (n = 2) [71,85], linear regression (n = 1) [69], multivariable regression (n = 1) [86], ordinal regression (n = 1) [85], and logistic regression (n = 4) [61,71,76,92].

Agreement or effect size measures were used nine times across the included studies. This included Cohen’s d (n = 2) [58,75], Cohen’s kappa (n = 1) [72], Bland–Altman (n = 2) [58,65], the Concordance index (n = 1) [68], confidence intervals (n = 1) [81], and root mean square deviation (n = 1) [63].

There were six instances in which the studies used validation/predictive modeling techniques. These included receiver operating characteristic curve analysis (n = 3) [76,77,95], cross-validation techniques (n = 2) [59,70], and Shapley additive index analysis (n = 1) [77].

Other statistical methods reported in this review included interquartile range (n = 1) [85], Chi-squared test (n = 2) [76,92], Fisher’s exact probability test (n = 1) [71], and the Shapiro–Wilk test (n = 1) [75].

### 3.6. Kinematic Features of Fall Risk

**Figure 2:**
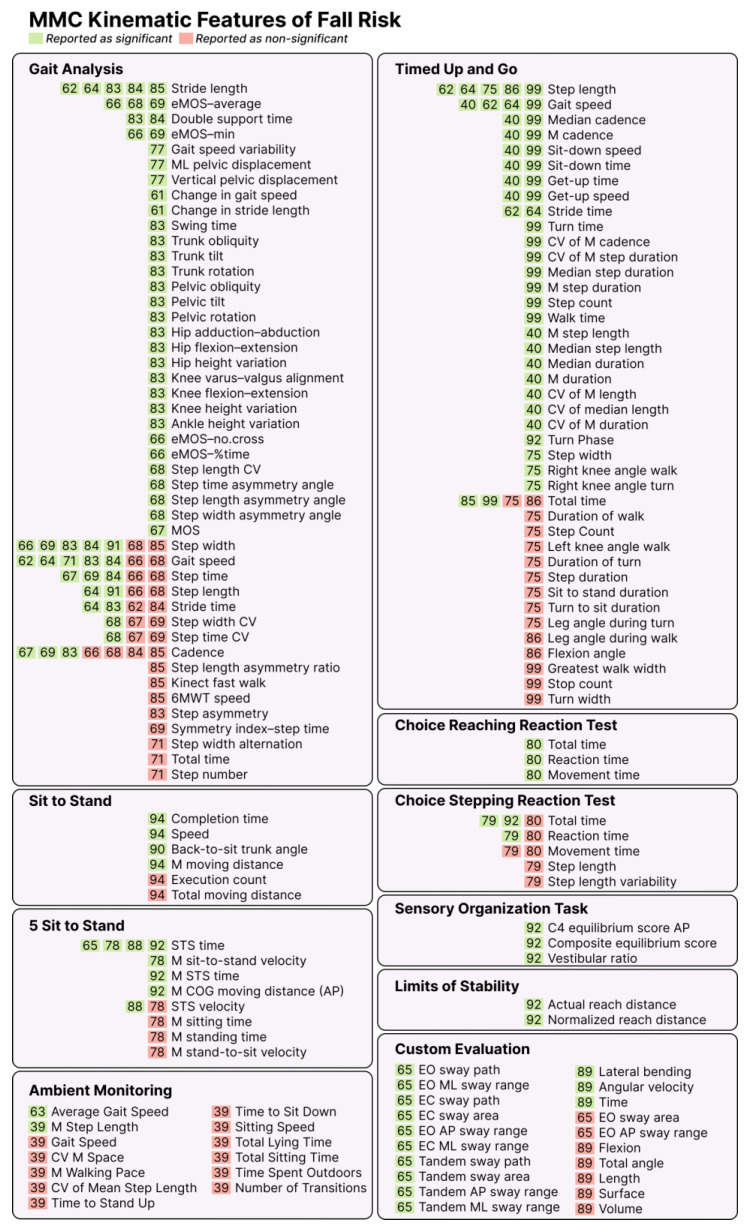
MMC features of fall risk. A summary of the biomarkers that are associated with fall risk. The number inside the square indicates the reference of the related study. AP: anterior–posterior, EO: eyes open, M: mean, EC: eyes closed, COG: center of gravity, STS: sit to stand, 6MWT: 6 min walk test, eMOS: estimated margin of stability, MOS: cargin of stability, CV: Coefficient of variability, Min: minimum.

Fall risk was assessed in 83% (n = 33) of the included studies [39,40,59,61,62,63,64,65,66,67,68,69,70,71,74,75,77,78,79,83,84,85,86,88,89,90,91,92,93,94,95,97]. Across these studies, a total of 285 kinematic features were identified across ten different MMC tasks or activities. Gait analysis was the most frequently used MMC task for fall risk (n = 12, 36%) [61,62,64,66,67,68,71,77,83,84,85,91]. The kinematic features associated with fall risk are summarized in Figure 2. The number of features identified per study ranged from 2 to 148, with a mean of 23.

Five unique studies reported significant associations between fall risk and gait speed [62,64,71,83,84], stride length [62,64,83,84,85], and step width [66,69,83,84,91], all derived from gait analysis. Among all the features examined, stride length was the only one that consistently demonstrated significant associations with fall risk. The remaining features, i.e., gait speed and step width, while significant in five studies, were also found to be non-significantly related to fall risk in two unique studies ([66,68] for gait speed and [68,85] for step width).

Step width, gait speed, and cadence were the most frequently reported kinematic features related to fall risk, each reported in seven unique studies (regardless of significance). Cadence was the most widely investigated non-significant parameter (n = 4) [66,68,84,85], although it was also found to be significantly associated with fall risk in nearly the same number of studies (n = 3) [67,69,83].

### 3.7. Kinematic Features of Frailty

**Figure 3:**
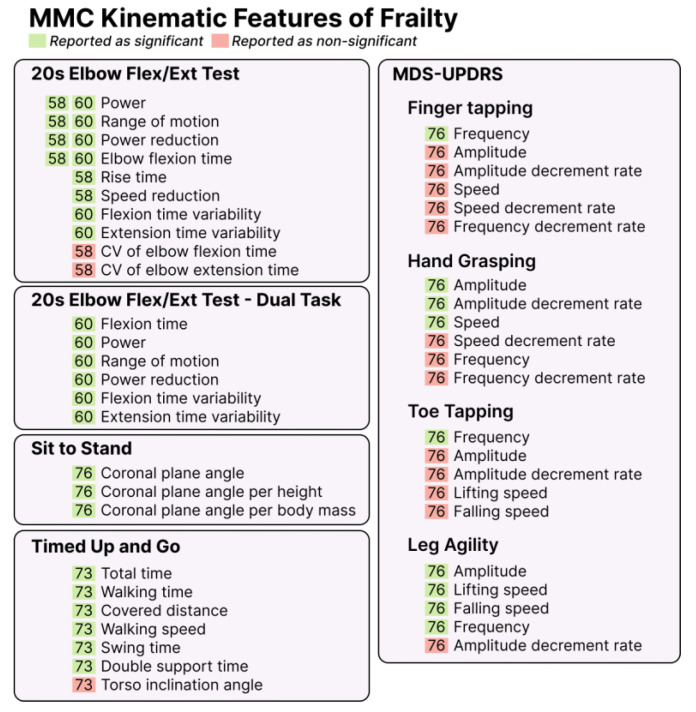
MMC features of frailty. MMC features associated with frailty. The number inside each square represents the reference. CV: coefficient of variability, MDS-UPDRS: Unified Parkinson’s Disease Ranking Scale, Flex-ext: flexion–extension, sec: second.

Frailty was assessed in seven studies (18%) [58,60,72,73,74,76,82,90], identifying 47 features across five different assessments. The kinematic features associated with frailty are presented in Figure 3. The number of features identified per study ranged from 3 to 22, with a mean of 9. There was limited consistency in the kinematic features investigated across the seven studies. The only assessment that appeared in two studies [58,60] was the 20 s arm flexion–extension task, with parameters measured during the task including power, degradation of speed, power reduction, range of motion, and elbow flexion time. These studies used different data collection methods, as listed in Table 3. There was no further overlap between any other studies that investigated frailty.

### 3.8. Feature Sets

Six studies investigated feature sets associated with fall risk [59,70,77,81,93,95], while two studies examined feature sets related to frailty [58,72]. These feature sets, composed of multiple interdependent features, are presented in Table 4. For studies reporting more than ten features, only the top ten are shown, based on the Shapley Additive Explanation values [98]. The remaining included features can be found in Supplementary Table S1.

### 3.9. Rates of Errors in Recording

Seven studies (18%) using Kinect video recordings excluded 68 of 1147 participants due to issues such as participant non-adherence to protocol, incomplete data capture, or failure to convert recordings into usable key point data [60,66,72,73,76,78,84]. The post hoc exclusion rates across these studies ranged from 2 to 15%, with a mean of 8%. These studies did not give specifics as to why there were poor rates of recording or data conversion.

An additional four studies (10%) using ambient monitoring with the Kinect device also reported reasons for participant exclusion [61,63,68,69], including obstruction of the legs by assistive devices (n = 2) [68,69] and difficulties distinguishing between participants with similar physical characteristics (n = 2) [61,63]. However, none reported the number of exclusions.

## 4. Discussion

This scoping review aimed to explore the kinematic features associated with fall risk or frailty. We identified 332 unique kinematic features derived from MMC technologies across 39 studies evaluating fall risk and/or frailty. Most studies (82%) focused on fall risk, 18% investigated frailty, and two studies (5%) examined both. While most studies involved older adults, one study included a healthy university student population [70]. Gait analysis was the most common assessment type (33% of the studies), and Microsoft Kinect was the predominant hardware platform (75% of the studies). Among the 332 features, 85% were related to fall risk, with minimal overlap in the reported parameters across studies. Gait speed, stride length, and step width were the most consistently reported parameters for fall risk, typically extracted during gait analysis or Timed Up and Go (TUG) tasks. In contrast, frailty-related features were less consistent, with only two studies reporting overlapping features: movement power, range of motion, degradation of speed, and elbow flexion time. This review is the first to synthesize the MMC kinematic features linked with frailty or fall risk (Figure 2 and Figure 3), providing a foundation for the design and advancement of future MMC technologies. By highlighting these features and the practical considerations for MMC integration into clinical practice, this review supports future research into MMC strategies for frailty and fall risk assessment in at-risk individuals.

Our gait-predominant fall risk findings align with previous reviews on digital mobility assessments using other movement sensors, like IMUs and depth cameras [49,99,100]. Often referred to as the sixth vital sign [101], gait speed is a well-established predictor of falls and mortality [102,103]. The prevalence of gait-based features in this review is consistent with IMU-focused reviews, including that by Ruiz-Ruiz et al., which emphasized gait speed, step time variability, and stride length as key fall risk markers [49]. Our review extends these findings to MMC systems. Mirroring our own findings, this review also emphasizes their use in broader functional assessments beyond gait alone, including lower-extremity strength and balance [49]. When comparing the number of features present for fall risk (Figure 2) versus frailty (Figure 3), it is evident that movement-based frailty identification has been less studied than fall risk. There were only four frailty features (power, reduction in power, elbow flexion time, and range of motion) that overlapped across studies, and all were drawn from just two studies. This indicates that frailty is currently under-studied, presenting a pressing need for more research in this area.

Beyond identifying features, our secondary aim was to evaluate practical and methodological aspects of MMC when used to assess frailty and fall risk. Here, we found considerable heterogeneity. Set-up procedures were inconsistently reported, with 15 studies omitting information about camera positioning and/or additional equipment requirements. Reported capture distances ranged widely from 0.8 to 6 m, and recording planes varied, though frontal-plane motion was the most common. Microsoft Kinect remained the predominant hardware platform, consistent with previous MMC reviews in rehabilitation [31], neurodegenerative disease [48], and other patient populations [104]. However, as Lam et al. [31] also observed, we noted a recent trend toward the use of commercially available devices, such as smartphones and digital cameras, suggesting a gradual shift from the historically dominant Kinect toward more scalable and accessible MMC tools.

Studies used between one and three devices to record motion, but limited reporting hindered the assessment of how device number influenced the accuracy of kinematic feature extraction. The few studies that did report accuracy relied exclusively on single-device setups [39,70,72,77,81,92,95]. Evidence from other contexts suggests that recording angle can substantially affect predictive accuracy of joint angles, particularly at the ankle [105,106]. More research is therefore needed to evaluate the impact of device number, camera angle, and recording plane on the accuracy of MMC-derived frailty and fall risk assessments. While the use of multiple devices may enhance predictive performance, the added complexity and resource requirements could create barriers to clinical implementation. Taken together, the variability in hardware choice, set-up procedures, and reporting highlights the lack of standardization in MMC-based frailty and fall risk research and limits comparability across studies.

Error rates and data exclusion were inconsistently reported across studies, with only 28% specifying reasons for removing data [60,61,63,66,68,69,72,73,76,78,84]. Clear documentation of when and why data are excluded is important to support reproducibility and guide algorithm refinement. Reporting of participant demographics was similarly limited. While age and sex were commonly reported, only one study described participant ethnicity [61]. Because MMC algorithms rely on predictive models trained on homogenous populations, limited demographic data hinders the evaluation of whether features are transferable to diverse groups [26]. As MMC-based assessments of frailty and fall risk advance, the systematic reporting of error and demographic information will strengthen the validity, equity, and real-world applicability of MMC tools.

While advances in research on kinematic features of frailty and fall risk highlight the potential of MMC, this review also identifies several methodological gaps that must be addressed before widespread clinical adoption is feasible. First, our review revealed limited standardization in device set-up and feature definitions, hindering cross-study comparability and external validity. Second, while the majority of studies reported the age (94%) and sex (84%) of participants, only one-third included BMI, and none stratified outcomes by demographic characteristics such as sex or age, both of which might impact MMC performance [107]. The absence of stratified analysis restricts our understanding of how these tools function across diverse populations. Third, while all studies compared MMC to validated clinical assessments (e.g., BBS or TUG) [54,55], some modified established clinical threshold cutoffs (e.g., gait speed reduced to <0.65 m/s [90], rather than the established cutoff of 0.8 m/s for community ambulation [108]) to optimize model performance. Although this may improve diagnostic accuracy in specific settings, it risks reducing generalizability and introducing algorithmic bias. Fourth, reporting of data quality was inconsistent. Only one-third of the studies explained participant exclusion or data loss, limiting reproducibility. These limitations echo those identified in prior MMC reviews [31,104] and reflect the early-stage nature of the field, where technical validation is often prioritized over population-level evaluation.

Translating MMC into real-world practice will require progress on these gaps as well as addressing additional practical barriers. At present, the only real-world implementation identified in this review involved ceiling-mounted Kinect devices [61,63,68,69], a configuration unlikely to be scalable in most clinical settings. Broader adoption will depend on the development of standardized assessment protocols, simplified set-up procedures, and solutions to technical challenges such as occlusion [26].

### 4.1. Future Directions

To strengthen the field and support clinical translation, future research should address key priorities, including (i) advancement towards a standardized set of MMC kinematic features for fall risk and frailty; (ii) a clear description of demographic factors and stratified analyses by factors, such as clinical condition, body mass index, or sex [26,107], in order to understand how these factors interact with MMC technology and enable tailored assessments; (iii) clear reporting on technical failures or drop-outs to understand how MMC functions in real-world settings and to improve algorithms; and (iv) enabling clinical implementation, through external validation and user-friendly interfaces.

### 4.2. Limitations

This scoping review has several limitations. Although the search strategy was comprehensive and developed with a research librarian, relevant studies may have been missed. In line with scoping review methodology, a formal risk of bias assessment was not conducted. The small number of studies on frailty, combined with heterogeneous protocols and inconsistent terminology, limited our ability to draw definitive conclusions about MMC’s role in frailty assessment. Furthermore, in line with the early stage of the research, most studies did not assess the same parameters. The inconsistent terminology used in the literature (e.g., “digital biomarker” vs. “kinematic feature” or “kinematic parameter,” “markerless motion capture” vs. “marker-free motion capture”) also presented a challenge, which may explain the large number of articles identified in the initial search.

## 5. Conclusions

This scoping review identified 332 features associated with fall risk and frailty. Of these, the most consistently identified as significant for fall risk are gait speed, stride length, and step width extracted during the TUG. The key frailty parameters identified include movement power, range of motion, degradation of speed, and elbow flexion time. This review highlights the diversity of MMC-derived kinematic features used in fall risk and frailty assessment. While fall risk has received more attention, both areas remain ripe for future development. The lack of standardization in feature selection, reporting, and demographic stratification limits comparability. Future research will benefit from harmonizing methods, improving reporting, and adequately reporting data loss. Further exploration of fall-risk and frailty MMC tools should occur in real-world settings, as this will be important in advancing these remote, accessible, and objective assessments that hold promise in supporting early detection and intervention across diverse populations.

## Figures and Tables

**Table 1:** A summary of the included studies and their populations.

Study Characteristics				Population Characteristics					
Study	Year	FR or F	N	Health Status	Sex M/F	Age Mean (SD)			Population at Risk of Falls or Frail n (%)
						O	C	P	
[84]	2020	FR	97	Patients with PD	49/48 ^a^	NR	67.8 (NR)	NR	6 (6.2)
[89]	2016	FR	22	Older adults	5/17 ^a^	82 (8)			22 (100)
[90]	2016	Both	60	Older adults	27/33	84 (5.2)	85.8 (5.2)	82.6 (4.7)	35 (58.3)
[85]	2019	FR	81	Stroke survivors	43/48	62.8 (12.3)	62.8 (12.3)	63.4 (15.6)	23 (28.4)
[91]	2023	FR	26	Older adults	NR	NR	66.1 (3.8)	66.2 (3.9)	13 (50)
[81]	2014	FR	79	Older adults	NR	NR	26 (5)	76 (10)	32 (40.5)
[60]	2024	F	65	Older adults	16/49	56.0 (18.7)	NR	NR	NR
[39]	2021	FR	30	Hospital in-patients	12/18	83.3 (8.5)	NR	NR	21 (70)
[40]	2019	FR	43	Older adults	16/27	83 (NR)	NR	NR	21 (48.8)
[96]	2017	FR	37	Hospital in-patients	14/23	83.6 (NR)	NR	NR	21 (56.8)
[78]	2015	FR	94	Older adults	28/66 ^a^	79.7 (6.4)	NR	NR	29 (30.9)
[79]	2014	FR	104	Older adults	34/70	80.7 (7.0)	NR	NR	68 (65.4)
[80]	2016	FR	94	Older adults	32/62 ^a^	80.6 (6.9)	NR	NR	19 (20.2)
[82]	2016	F	30	Older adults	5/25	75.6 (7.5)	NR	NR	17 (56.8)
[70]	2023	FR	6	University students	NR	NR	NR	NR	NR
[59]	2014	FR	12	Older adults	NR	NR	NR	NR	7 (58.3)
[71]	2011	FR	30	Patients with PD	14/16 ^a^	68.3 (7)	NR	NR	15 (50)
[92]	2024	FR	106	Older adults	0/106	NR	74.2 (5.1)	76.6 (5)	22 (20.8)
[83]	2019	FR	437	Stroke survivors	224/213 ^a^	NR	48.3 (16.1)	43.3 (18.6)	18 (4.1)
[93]	2024	FR	65	MMU-FRiP: non-fallers Mendeley: fallers	MMU-FRiP: 18/3 Mendeley: 7/37 ^a^	NR	NR	70.0 (8.6)	44 (67.7)
[66]	2020	FR	52	Older adults	28/24	76.3 (8)	NR	NR	28 (53.8)
[67]	2022	FR	14	Older adults	3/11	86.7 (6.2)	NR	NR	NR
[68]	2021	FR	51	Patients with dementia	23/28	76.3 (7.9)	NR	NR	51 (100)
[72]	2024	F	417	Patients with heart failure	222/194	VC: 82.5 (5.2) DC: 82.3 (4.9)	NR	NR	417 (100)
[69]	2020	FR	32	Dementia	18/11		75.5 (8.6)	78.3 (8.9)	17 (54.8)
[61]	2017	FR	23	Older adults	7/16	85.2 (NR)			13 (56.5)
[62]	2015	FR	19	Older adults	9/10 ^a^	87 (NR)			NR
[64]	2013	FR	32	Older adults	7/8 ^a^		56.5 (11.5)	87.5 (7.9)	NR
[88]	2020	FR	37	Older adults	NR		28.3 (6.8)	67.2 (6.7)	16 (43.2)
[63]	2015	FR	16	Older adults	7/9	85.8 (8.0)	NR	NR	NR
[94]	2020	FR	15	Stroke survivors	13/2	58.6 (8.7)	NR	NR	NR
[65]	2019	FR	30	Bone clinic patients	0/30	NR	74.5 (6.2)	80.8 (9.2)	10 (33.3)
[73]	2019	F	402	Older adults	136/266 ^a^	73.7 (7.5)	NR	NR	90 (22.4)
[95]	2018	FR	224	Patients with neurological disorders	144/80	67.5 (14)	NR	NR	45 (20.1)
[86]	2015	FR	30	Stroke survivors	21/9	68 (15)	NR	NR	NR
[75]	2024	FR	41	Older adults	5/36	NR	77.4 (5.3)	82.0 (7.4)	15 (36.6)
[76]	2023	F	52	Patients with PD	24/28	65.5 (8.9)	65.5 (NR)	69 (NR)	32 (61.5)
[58]	2020	F	21	Patients with COPD	NR	67.8 (10.7)	NR	NR	NR
[77]	2023	FR	46	Older adults	15/31	71.4 (5.11)	NR	NR	10 (21.7)

^a^: only gender reported without sex specified, F: frailty, FR: fall risk, PD: Parkinson’s disease, COPD = chronic obstructive pulmonary disease, O: overall, C: control, P: participant, NR: not reported, N: number, M: male, F: female, SS: sample size, M: mean, SD: standard deviation.

**Table 2:** Technology characteristics. A summary of the technology characteristics of the included studies.

	Hardware				MMC Set-Up			
Study	Device	n of Devices	Set-Up	Additional Equipment	Algorithm	Key Points	Features	Features or Feature Set?
Alvarez 2020 [84]	Kinect v2	1	H: 0.8 m D: 1.5 Frontal plane	WBB, table, laptop	NR	NR	20	Features
Bonnechère 2016 [89]	Microsoft Kinect	1	Frontal plane	WBB, table, display screen	Kinect-based skeletal tracking	NR	8	Features
Bourrelier 2016 [90]	Kinect	1	D: 2.5 m A: 20 degrees Sagittal plane	Chair with armrests	Kinect-based skeletal tracking	NR	2	Features
Bower 2019 [85]	Microsoft Kinect	1	D: 1.8–4.0 m	Table, laptop	Kinect-based skeletal tracking	NR	9	Features
Camargos 2023 [91]	Kinect	3	NR	Leap motion controller	NR	NR	20	Features
Colagiorgio 2014 [81]	Microsoft Kinect	1	D: 2 m Frontal plane	NR	NR	NR	80	Features
Dehghan Rouzi 2024 [60]	Smartphones or Tablet cameras	1	Sagittal plane	NR	Google’s MediaPipe	32	14	Features
Dubois 2017 [96]	Microsoft Kinect	1	D: 4 m Sagittal plane	Stopwatch	Custom algorithm	NR	21	Features
Dubois 2019 [40]	Kinect	1	D: 4 m H: 1.7 m A: 20 degrees Sagittal plane	NR	Custom algorithm	NR	17 features Set of 1–3 features	Feature set + features
Dubois 2021 [39]	Kinect v2	1	A: 20 degrees	Tripod	Custom algorithm	NR	15 features Set of 2 features	Feature set + features
Ejupi 2014 [79]	Microsoft Kinect	1	D: 2 m H: 0.8 m Frontal plane	TV, tripod/table	Kinect-based skeletal tracking	NR	6	Features
Ejupi 2015 [78]	Microsoft Kinect	1	D: 2 m H: 0.8 m Frontal plane	Monitor	Kinect-based skeletal tracking	NR	5	Features
Ejupi 2016 [80]	Microsoft Kinect	1	D: 2 m H: 0.8 m Frontal plane	TV screen	Kinect-based skeletal tracking	NR	8	Features
Gianaria 2016 [82]	Microsoft Kinect	1	D: 4 m H: 2 m	NR	Custom algorithm	25	7	Features
Kamahori 2023 [70]	Intel RealSense D435 2D camera	1	D: 3.25 m H: 1.1 m	Tripod	Custom algorithm	NR	Set of 5 features	Feature sets
Kargar 2014 [59]	Microsoft Kinect	1	D: 3.5 m H: 1.2 m	Table, chair	Kinect-based skeletal tracking	20	5	Features
Kataoka 2011 [71]	Unspecified camera	1	NR	NR	Manual labeling	NR	21	Features
Kim 2024 [92]	Kinect	1	D: 3.2 m H: 0.7 m Frontal plane	Table, chair, cone, balance pad	Kinect-based skeletal tracking	25	66	Features
Latorre 2019 [83]	Kinect v2	1	D: 6 m	NR	Custom algorithm	25	23	Features
Lim 2024 [93]								
Mehdizadeh 2020 [66]	Kinect V2	1	Ceiling mount	RFID tags	Kinect-based skeletal tracking	NR	30	Features
Mehdizadeh 2021 [68]	Kinect	1	Ceiling mount	RFID tags	Kinect-based skeletal tracking	NR	12	Features
Mehdizadeh 2022 [67]	Motorola Moto G5 Play cell phones	2	D: 1.1–2 m	IMUs	Alphapose, OpenPose, and Detectron	NR	24	Features
Mizuguchi 2024 [72]	iPod touch, seventh generation	1	D: 4 m H: 1 m	Tripod, floor markers, chair	OpenPose	25	Set of 45 features	Feature sets
Ng 2020 [69]	Kinect V2	1	Ceiling mount	NR	Openpose	13	7	Features
Phillips 2017 [61]	Microsoft Kinect	1	NR	NR	Custom algorithm	NR	3	Features
Rantz 2013 [64]	Microsoft Kinect	1	Ceiling mount	NR	Custom algorithm	NR	3	Features
Rantz 2015 [62]	Microsoft Kinect	1	Ceiling mount	NR	Custom algorithm	NR	3	Features
Shukla 2020 [88]	Kinect	2	D: 2.3 m Frontal plane	NR	Custom algorithm	15	2	Features
Stone 2015 [63]	Microsoft Kinect	1	Ceiling mount	NR	Kinect-based skeletal tracking	NR	1	Features
Sun 2019 [65]	Microsoft Kinect	1	D: 2 m H: 1 m	PC-based computer and a display screen	Kinect-based skeletal tracking	NR	13	Features
Sun 2020 [94]	Xbox 360 Kinect	1	Frontal plane	Display	Unity3D software	NR	5	Features
Takeshima 2019 [73]	Microsoft Kinect		D: 3 m H: 0.1 m	Tripod, laptop	Kinect-based skeletal tracking	25	3	Features
Tripathy 2018 [95]	Kinect Xbox 360 (Kinect 1) and Kinect Xbox One (Kinect 2)	2	D: 3 m Frontal plane	NR	Custom algorithm	20	Set of 7 features	Feature sets
Vernon 2015 [86]	Kinect Xbox 360	1	Frontal plane	Table, chair	Custom algorithm	7	7	Features
Wang 2024 [75]	Microsoft Kinect	1	NR	Chair, tripod	Kinect-based skeletal tracking	25	142	Features
Xie 2023 [76]	Azure Kinect	1	D: 1.2–2.2 m H: 1 m	Table, chair	Custom algorithm	32	22	Features
Zahiri 2020 [58]	Samsung Galaxy Tablet	1	Sagittal plane	Tripod, IMU	OpenPose	3	20	Features
Zhang 2023 [77]	Azure Kinect	1	D: 0.8 m Frontal plane	IMUs	Kinect-based skeletal tracking	32	8	Features

D: distance from subject, H: height of camera, IMUs: Inertial measurement units, NR: not reported, n: number, WBB: Wii Balance Board, RFID: radio-frequency identification, PC: personal computer, TV: television.

**Table 3:** Assessment characteristics. A summary of assessment characteristics of the included studies.

	Frailty/Fall Risk Reference Assessment (Non-MMC)			
Study	Type	Reference Measure	Administered By	MMC Task/Activity
Alvarez 2020 [84]	Clinical	POMA	Neurologist	Gait analysis
Bonnechère 2016 [89]	Clinical	Tinetti, BBS, TUG, 30 s STS	Clinical evaluation	Video game
Bourrelier 2016 [90]	Clinical	TUG and gait speed	PT	STS
Bower 2019 [85]	Clinical	Step test, TUG, prospective fall monitoring	PT and EP	Gait analysis
Camargos 2023 [91]	Self-report	Fall history	Self-report	Gait analysis
Colagiorgio 2014 [81]	Clinical	Tinetti test	Clinician	Tinetti test
DehghanRouzi 2024 [60]	Clinical	Frailty meter assessment protocol	Research staff	20 s elbow flexion and extension test
Dubois 2017 [96]	Clinical	TUG	Healthcare professional	TUG
Dubois 2019 [40]	Clinical	TUG	Healthcare professional	TUG
Dubois 2021 [39]	Clinical	Tinetti test, TUG	PT	Ambient monitoring
Ejupi 2014 [79]	Self-report	Fall history	Self-report	CSRT
Ejupi 2015 [78]	Clinical	5STS, fall history	Research staff	5STS
Ejupi 2016 [80]	Clinical	PPA and prospective fall reporting	Research staff	Choice reaction times
Gianaria 2016 [82]	Self-report	TUG, TFI	Self-report	TUG
Kamahori 2023 [70]	Clinical	Tinetti test	NR	Balance task
Kargar 2014 [59]	Clinical	Get up and go task	Physician	TUG
Kataoka 2011 [71]	Self-report	Fall history	Self-report	Gait analysis
Kim 2024 [92]	Self-report	Prospective fall monitoring	Self-report	TUG
Latorre [83]	Clinical	BBS	NR	Gait analysis
Lim 2024 [93]	Clinical	POMA and JHFRAT	Unspecified	TUG
Mehdizadeh 2020 [66]	Self-report	Prospective fall monitoring	Research Staff	Gait analysis
Mehdizadeh 2021 [68]	Self-report	Prospective fall monitoring	Research Staff	Gait analysis
Mehdizadeh 2022 [67]	Clinical	BBS, POMA, TUG	Research staff	Gait analysis
Mizuguchi 2024 [72]	Clinical	Clinical frailty scale	Cardiologists	Gait analysis
Ng 2020 [69]	Self-report	Prospective fall monitoring, POMA	Research staff and healthcare professional	Ambient monitoring
Phillips 2017 [61]	Self-report	Prospective fall monitoring	Self-report	Gait analysis
Rantz 2013 [64]	Clinical	BBS, TUG, SPPB, SLS, HGS, FAP	Research staff	Gait analysis
Rantz 2015 [62]	Clinical	HGS, FRT, BBS, TUG, SPPB, SLS	Research staff	Gait analysis
Shulka 2020 [88]	Clinical	5STS	“Expert”	5STS
Stone 2015 [63]	Clinical	TUG, HGS, BBS, MDRT	Research staff	Ambient monitoring
Sun 2019 [65]	Clinical	TUG, BBS, FES	Research staff	Video game
Sun 2020 [94]	Clinical	Biodex balance system and TUG	PT and research staff	Video game
Takeshima 2019 [73]	Clinical	Functional independence measure	PT	STS
Tripathy 2018 [95]	Clinical	BBS	NR	SLS
Vernon 2015 [86]	Clinical	Step test, TUG, FRT	Assessor	TUG
Wang 2024 [75]	Clinical	BBS, TUG	NR	TUG
Xie 2023 [76]	Clinical	Fried’s frailty criteria	Movement disorder specialist	MDS-UPDRS-III
Zahiri 2020 [58]	Clinical	Fried’s frailty criteria	Validated IMU assessment	20 s elbow flexion and extension test
Zhang 2023 [77]	Self-report	Fall history	Self-report	Gait analysis

NR: not reported, TUG: Timed Up and Go, BBS: Berg Balance Scale, FRT: Functional Reach Test, HGS: habitual gait speed, SLS: single-leg stance, SPPB: short physical performance battery, FAP: functional ambulation profile, MDS-UPDRS-III: Unified Parkinson’s Disease Ranking Scale part three, POMA: Tinetti Performance Oriented Mobility Assessment, JHFRAT: John Hopkins Fall Risk Assessment Tool, TFI: Tilburg frailty indicator, STS: sit to stand, PT: physiotherapist, EP: exercise physiologist, IMU: inertial measurement unit, 5STS: five times sit to stand, FES: Falls Efficacy Scale.

**Table 4:** Feature sets. Only studies that examined a defined set of features are included in this table. The top ten features are reported here, as ranked by the SHAP index.

Reference	Fall Risk or Frailty?	MMC Task/Activity	# of Features	Top 10 Features
Colagiorgio 2014 [81]	Fall risk	Tinetti test	8	Maximum amplitude of chest pitch, velocity of the steps, chest pitch during standing from chair, standing eyes open-Ks (postural control), standing eyes open SD, sit down chest pitch, standing eyes open mean velocity, sternal nudge changes in txc (postural control)
Kamahori 2023 [70]	Fall risk	Clinical test of sensory interaction and balance	5	M displacement of the center of gravity, the instantaneous max displacement of the center of gravity, the M displacement before and after the center of gravity, the instantaneous max displacement before and after the center of gravity, the variance in the arm swing width
Kargar 2014 [59]	Fall risk	TUG	7	Number of steps, duration of each step, number of steps in turning phase, distance between two elbows, angle between the legs, right and left knee angles
Lim 2024 [93]	Fall risk	TUG	4	Ave step time, cadence, ave stride time, ave stance time
Mizuguchi 2024 [72]	Frailty	Gait analysis	45	Gait speed, total gait time, spine angle in frontal walking, stance phase time, elbow angle (median), ankle swing speed (max), heel angle (min), trajectory of the ankle distance (max), ankle lift speed (max), cornering time… *
Tripathy 2018 [95]	Fall risk	BBS	7	Zero crossing rate, SLS duration, spectral entropy, disease, gender, fall history, postural deviation
Zahiri 2020 [58]	Frailty	20 s arm flexion–extension test	5	Range of motion, percentage of decline in power, flexion time, flexion time variability, extension time variability
Zhang 2023 [77]	Fall risk	Gait analysis	20	Step frequency, BMI, gait cycle variability, hypertension, eye diseases, dyslipidemia, age, CV disease, diabetes, stride CV… *

M: mean, max: maximum, ave: average, SD: standard deviation, min: minimum, SLS: single-leg stance. * The full feature set can be found in Supplementary Table S1.

## Supplementary Materials

The following supporting information can be downloaded at https://www.mdpi.com/article/10.3390/s25185741/s1. Table S1: Extended feature sets.
