# Supplementary material for: Markerless Motion Capture Parameters Associated with Fall Risk or Frailty: A Scoping Review
Source: Sensors (Basel). 2025 Sep 15;25(18):5741. doi: 10.3390/s25185741 (PMC12473936; doi:10.3390/s25185741)
Supplement: Supplementary file 1 [file sensors-25-05741-s001.zip › Full Feature Sets.pdf]

Supplementary Table S1. Extended Feature sets

Reference	Fall risk or frailty?	Assessment	# of features	Top 10 features
Mizuguchi 2024 [52]	Frailty	Gait analysis	45	Gait speed, Total gait time, Spine angle in frontal walking (SD), Stance phase time, Elbow angle (median), Ankle swing speed (max), Heel angle (min), Trajectory of the ankle distance (max), Ankle lift up speed (max), Cornering time, Trajectory of the ankle distance (mean), Wrist momentum, Stance phase, Hip swing amplitude (max), Slow speed time, Spine angle in stand up (mean), Ankle swing acceleration (max), Spine angle in stand up (min), Ankle swing amplitude (min), Wrist swing speed (max), Hip swing amplitude (SD), Swing phase time, Shoulder swing amplitude (mean), Step width, Spine angle in frontal walking (mean), Ankle momentum, Ankle lift up distance (max), BV12, Big toe swing amplitude (min), Bilateral ankle distance (mean), Hip swing amplitude (min), Wrist swing amplitude (max), Wrist swing amplitude (mean), Shoulder slope (variance), Angle of spine and pelvis (variance), Wrist swing amplitude (variance), Shoulder swing amplitude (min), X coordinates of the right wrist (mean), Wrist swing amplitude (SD), Trajectory of the knee distance (mean), Spine angle in stand up (max), Pelvis bone slope (max), Hip joint angle (variance), Arm swing amplitude (max), X coordinate of the right ankle (variance), Bilateral ankle distance (variance).
Zhang 2023 [57]	Fall risk	Gait analysis	20	Step frequency, BMI, period_cv, Hypertension, Eye diseases, Dyslipidemia, Age, Cardiovascular disease, Diabetes, stride_cv, Cerebrovascular disease, Gender, Gait period, Stride, Pace, Swing phase, alcohol consumption, Double support period, physical activity, Osteoarthritis.

Supplementary Table S1 legend. BMI: Body mass index, period_cv: Gait period variability, Stride_cv: Stride variability, min: minimum, SD: standard deviation, max: maximum, BV12: Effect of the knee joint on the first factor of principal component analysis of the hip, knee, and ankle joints
