# Supplementary material for: Markerless Motion Capture Parameters Associated with Fall Risk or Frailty: A Scoping Review
Source: Sensors (Basel). 2025 Sep 15;25(18):5741. doi: 10.3390/s25185741 (PMC12473936; doi:10.3390/s25185741)
Supplement: Supplementary file 1 [file sensors-25-05741-s001.zip › Search Strategy.pdf]

Databases to include: Medline, Embase, CINAHL, Scopus
Conference abstracts: No
Date - no date limit
Language limits - English only

Ovid MEDLINE(R) ALL <1946 to October 24, 2024>

https://login.ezproxy.library.ualberta.ca/login?url=http://ovidsp.ovid.com/ovidweb.cgi?T=JS&NEWS=N&PAGE=main&SHAREDSEARCHID=5ZQHGVUtaBCiPKwK80s9Ej83Rvwmj0MZg0FH4OOSr5D3iIp5oaDlM7V3Dc0nATU6p

1 ("motion capture" or mocap or "motion tracking" or "motion analysis").mp. 17383
2 ((markerless or marker-less) and (video or camera or smartphone* or "smart phone*" or iphone* or "cell* phone*")).mp. 439
3 ((markerless or marker-less or "video recording*" or "record* video*" or "video tap*" or videotap* or "standard video*" or "simple video*" or "regular video*" or "single video*" or "ordinary video*" or "single camera" or "depth camera" or smartphone* or "smart phone*" or "phone camera*" or "camera phone*" or "cell* phone*" or video-based or 2D or 2-D or "2 dimensional" or "two dimensional" or "pose estimation" or openpose or alphapose or deepcut or "deep learning" or ai or "machine learning") and ("motion capture" or mocap or "motion analys*" or "kinematic data" or (track* adj2 (body or movement or motion or kinematic*)))).mp. 3747
4 ((markerless or marker-less or "video recording*" or "record* video*" or "standard video*" or "simple video*" or "regular video*" or "single video*" or "ordinary video*" or "single camera" or "depth camera" or "phone camera*" or "camera phone*" or video-based or videotape* or "video tap*" or "pose estimation" or openpose or alphapose or deepcut) and ((assess* or analy* or evaluat* or measur* or parameter* or impair* or deviat* or patholog* or dysfunction*) adj3 (movement or motor or motion or gait or balance or postur* or kinematic*))).mp. 4346
5 ("Leap motion" or Pose2Sim or Kinect or KinectV2 or DeepLabCut or "Deep lab cut" or Trazer or Lidar or "RGB-D camera" or Freemocap or "Theia markerless" or Theia3D or "Theia 3D" or Captury or CapturyLive or MoveAI or "move AI" or "iPi soft" or "move.ai or simi shape 3d" or "simi motion" or ((vicon or qualisys) and markerless) or "biomotion lab" or "radical motion" or OpenCap or KinaTrax).mp. 7402
6 1 or 2 or 3 or 4 or 5 28054
7 ((Fall or falls or falling) adj3 (risk or risks or likelihood or assess* or evaluat* or test or determin* or screen*)).mp. 19649
8 frail*.mp. 46831
9 ("functional evaluation*" or "functional assessment*" or "Functional analys*" or "Fugl Meyer Assessment*" or "stepping in place" or "balance test*" or "balance assessment*" or "stepping test*" or "timed up and go" or "berg balance" or "edmonton frail scale" or "fried frailty" or "frailty index" or "comprehensive giereatric assessment" or "clinical frailty scale" or "pictorial fit-to-frail scale" or "hierarchical assessment of balance and mobility" or "IoRN" or "SPARRA" or "eFI" or "FiND" or "Easycare" or "think frailty" or "gérontopôle" or "APOP" or "ISAR" or "PRISMA 7" or "groningen frailty index" or "frailty phenotype" or "FRESH-screening" or "TUGT" or "frail scale" or "falls screening" or "MBESS" or "BESTest" or "Mini-BEST" or "MD-UPDRS" or MDS-UPDRS or "nine-hole peg" or "9-hole peg" or UPDRS or UPRDS or "box and block" or "sit to stand" or "one leg stance test" or "forward functional reach" or "functional reach test" or "gait assessment" or "functional gait assessment" or "Tinetti gait assessment" or "gait assessment and intervention tool" or "dynamic gait index" or "up and go test" or "activities specific balance confidence scale" or "gait and balance scale" or "functional ambulation classification scale").mp. 564973

10 ((parameter* or impair* or deviat* or patholog* or dysfunction*) adj3 (movement or motor or motion or gait or balance or postur* or kinematic)).mp. 58459
11 (gait adj5 freez*).mp. 2017
12 7 or 8 or 9 or 10 or 11 671759
13 exp Dementia/ or exp cardiovascular diseases/ or exp Parkinsonian Disorders/ or exp Multiple Sclerosis/ or Amyotrophic Lateral Sclerosis/ or exp Muscular Dystrophies/ or exp Renal Insufficiency, Chronic/ or liver cirrhosis/ or liver cirrhosis, alcoholic/ or liver cirrhosis, biliary/ or exp brain injuries, traumatic/ or exp brain injury, chronic/ or exp Pulmonary Disease, Chronic Obstructive/ or exp Neurodegenerative Diseases/ 3642179
14 (dementia or "heart failure" or "heart disease" or "cardiac disease*" or "cardiovascular disease" or hypertension or stroke or poststroke or transient ischemic attack* or ischaemic attack* or cerebrovascular accident* or alzheimer* or parkinson* or "multiple scleros*" or "amyotrophic lateral sclerosis" or "motor neuron disease" or huntington* or "muscular dystroph*" or "chronic kidney diseas*" or "chronic renal insufficiency" or "chronic kidney injur*" or "kidney failure" or "end stage kidney disease" or cirrhosis or "traumatic brain injur*" or "acquired brain injur*" or (chronic adj2 ("lung disease" or "pulmonary disease*")) or COPD or "chronic obstructive pulmonary" or ((degenerative or neurodegenerative or neurological) adj4 (diseas* or illness* or disorder* or condition*))).mp. 2625327
15 exp aged/ or ("over 65" or "65 and over" or "65 or over" or "65+ years" or frail* or retired or sarcopeni* or elder* or old* old or old age or centenarian* or nonagenarian* or octogenarian* or septuagenarian* or aging or (senior* not ((high school or university or college) adj3 senior*)) or gerontolog* or geriatric* or veteran* or post-menopaus* or postmenopaus* or mature adult* or mature patient* or mature individual* or mature resident* or aged adult* or aged patient* or aged individual* or aged resident* or aged donor* or aged population* or aged care or nursing home resident* or nursing home patient* or (older adj2 (people or person* or client* or resident* or adult* or patient* or individual* or donor* or population* or women or men))).mp. 4254743
16 13 or 14 or 15 7576570
17 6 and 12 and 16 1771
18 limit 17 to animals 51
19 limit 18 to humans 11
20 17 not (18 not 19) 1731
21 (animal-model* or pigeon* or rat or rats or pig or pigs or porcine or mouse or mice or murine or hamster or hamsters or cats or feline or dog or dogs or canine or bovine or sheep or primate* or monkey* or zebra* or drosophila* or in-vivo or cadaver*).ti,bt,kf. 2576204
22 20 not 21 1715
23 (exergam* or "gamified exercis*" or swim).ti,bt,kf. 4079
24 22 not 23 1674
25 ((child or children or infant* or neonat* or adolescen* or teen* or youths or pediatric* or paediatric*) not adult*).ti,kf. 1578960
26 24 not 25 1644
27 limit 26 to english language 1615

Embase <1974 to 2024 October 24>(Ovid interface)
Date searched: Oct 25, 2024

https://login.ezproxy.library.ualberta.ca/login?url=http://ovidsp.ovid.com/ovidweb.cgi?T=JS&NEWS=N&PAGE=main&SHAREDSEARCHID=22G8lYVfYmkj24Yvk8ZJxWnxET7gXt4RW2qzWD1VWmRklljxnqhzE8B6Aqiea3j46

1 motion analysis system/ or motion capture/ 12661
2 ("motion capture" or mocap or "motion tracking" or "motion analysis").mp. 28561
3 ((markerless or marker-less) and (video or camera or smartphone* or "smart phone*" or iphone* or "cell* phone*")).mp. 584
4 ((markerless or marker-less or "video recording*" or "record* video*" or "video tap*" or videotap* or "standard video*" or "simple video*" or "regular video*" or "single video*" or "ordinary video*" or "single camera" or "depth camera" or smartphone* or "smart phone*" or "phone camera*" or "camera phone*" or "cell* phone*" or video-based or 2D or 2-D or "2 dimensional" or "two dimensional" or "pose estimation" or openpose or alphapose or deepcut or "deep learning" or ai or "machine learning") and ("motion capture" or mocap or "motion analys*" or "kinematic data" or (track* adj2 (body or movement or motion or kinematic*)))).mp. 4960
5 ((markerless or marker-less or "video recording*" or "record* video*" or "standard video*" or "simple video*" or "regular video*" or "single video*" or "ordinary video*" or "single camera" or "depth camera" or "phone camera*" or "camera phone*" or video-based or videotape* or "video tap*" or "pose estimation" or openpose or alphapose or deepcut) and ((assess* or analy* or evaluat* or measur* or parameter* or impair* or deviat* or patholog* or dysfunction*) adj3 (movement or motor or motion or gait or balance or postur* or kinematic*))).mp. 4152
6 ("Leap motion" or Pose2Sim or Kinect or KinectV2 or DeepLabCut or "Deep lab cut" or Trazer or Lidar or "RGB-D camera" or Freemocap or "Theia markerless" or Theia3D or "Theia 3D" or Captury or CapturyLive or MoveAI or "move AI" or "iPi soft" or "move.ai or simi shape 3d" or "simi motion" or ((vicon or qualisys) and markerless) or "biomotion lab" or "radical motion" or OpenCap or KinaTrax).mp. 7151
7 1 or 2 or 3 or 4 or 5 or 6 38166
8 fall risk/ or fall risk assessment/ 8283
9 ((Fall or falls or falling) adj3 (risk or risks or likelihood or assess* or evaluat* or test or determin* or screen*)).mp. 30781
10 frail*.mp. 72532
11 ("functional evaluation*" or "functional assessment*" or "Functional analys*" or "Fugl Meyer Assessment*" or "stepping in place" or "balance test*" or "balance assessment*" or "stepping test*" or "timed up and go" or "berg balance" or "edmonton frail scale" or "fried frailty" or "frailty index" or "comprehensive giereatric assessment" or "clinical frailty scale" or "pictorial fit-to-frail scale" or "hierarchical assessment of balance and mobility" or "IoRN" or "SPARRA" or "eFI" or "FiND" or "Easycare" or "think frailty" or "gérontopôle" or "APOP" or "ISAR" or "PRISMA 7" or "groningen frailty index" or "frailty phenotype" or "FRESH-screening" or "TUGT" or "frail scale" or "falls screening" or "MBESS" or "BESTest" or "Mini-BEST" or "MD-UPDRS" or MDS-UPDRS or "nine-hole peg" or "9-hole peg" or UPDRS or UPRDS or "box and block" or "sit to stand" or "one leg stance test" or "forward functional reach" or "functional reach test" or "gait assessment" or "functional gait assessment" or "Tinetti gait assessment" or "gait assessment and intervention tool" or "dynamic gait index" or "up and go test" or "activities specific balance confidence scale" or "gait and balance scale" or "functional ambulation classification scale").mp. 786665
12 ((parameter* or impair* or deviat* or patholog* or dysfunction*) adj3 (movement or motor or motion or gait or balance or postur* or kinematic)).mp. 155069
13 (gait adj5 freez*).mp. 4089
14 8 or 9 or 10 or 11 or 12 or 13 1003870
15 exp dementia/ 479792

16 exp cardiovascular disease/ 5339819
17 exp Parkinson disease/ 206571
18 exp multiple sclerosis/168221
19 amyotrophic lateral sclerosis/ or motor neuron disease/ 61953
20 exp chronic kidney failure/ 174910
21 exp liver cirrhosis/ 203456
22 traumatic brain injury/ or chronic traumatic encephalopathy/ 73186
23 brain injury/ or acquired brain injury/ 103437
24 chronic obstructive lung disease/ 191507
25 degenerative disease/ or exp hereditary neurodegenerative disease/ 91767
26 (dementia or "heart failure" or "heart disease" or "cardiac disease*" or "cardiovascular disease" or hypertension or stroke or poststroke or transient ischemic attack* or ischaemic attack* or cerebrovascular accident* or alzheimer* or parkinson* or "multiple scleros*" or "amyotrophic lateral sclerosis" or "motor neuron disease" or huntington* or "muscular dystroph*" or "chronic kidney diseas*" or "chronic renal insufficiency" or "chronic kidney injur*" or "kidney failure" or "end stage kidney disease" or cirrhosis or "traumatic brain injur*" or "acquired brain injur*" or (chronic adj2 ("lung disease" or "pulmonary disease*")) or COPD or "chronic obstructive pulmonary" or ((degenerative or neurodegenerative or neurological) adj4 (diseas* or illness* or disorder* or condition*))).mp. 4608994
27 aged/ or aged hospital patient/ or frail elderly/ or institutionalized elderly/ or very elderly/ 3970539
28 ("over 65" or "65 and over" or "65 or over" or "65+ years" or frail* or retired or sarcopeni* or elder* or old* old or old age or centenarian* or nonagenarian* or octogenarian* or septuagenarian* or aging or (senior* not ((high school or university or college) adj3 senior*)) or gerontolog* or geriatric* or veteran* or post-menopaus* or postmenopaus* or mature adult* or mature patient* or mature individual* or mature resident* or aged adult* or aged patient* or aged individual* or aged resident* or aged donor* or aged population* or aged care or nursing home resident* or nursing home patient* or (older adj2 (people or person* or client* or resident* or adult* or patient* or individual* or donor* or population* or women or men))).mp. 2138189
29 15 or 16 or 17 or 18 or 19 or 20 or 21 or 22 or 23 or 24 or 25 or 26 or 27 or 28 10716332
30 7 and 14 and 29 3614
31 limit 30 to conference abstracts 1015
32 30 not 31 2599
33 limit 32 to animal studies 63
34 limit 33 to human 3
35 32 not (33 not 34) 2539
36 (animal-model* or pigeon* or rat or rats or pig or pigs or porcine or mouse or mice or murine or hamster or hamsters or cats or feline or dog or dogs or canine or bovine or sheep or primate* or monkey* or zebra* or drosophila* or in-vivo or cadaver*).ti,bt,kf. 2869567
37 ((child or children or infant* or neonat* or adolescen* or teen* or youths or pediatric* or paediatric*) not adult*).ti,kf. 1878871
38 (exergam* or "gamified exercis*" or swim*).ti,bt,kf. 20094
39 35 not (36 or 37 or 38) 2402
40 limit 39 to english language 2347

CINAHL Plus with Full Text (EBSCOhost interface)
Date searched: Oct 25, 2024
Results:704

S1 "motion capture" OR mocap OR "motion tracking" OR "motion analysis" OR ((markerless OR marker-less) AND (video OR camera OR smartphone* OR "smart phone*" OR iphone* OR "cell* phone*")) OR ((markerless OR marker-less OR "video recording*" OR "record* video*" OR "video tap*" OR videotap* OR "standard video*" OR "simple video*" OR "regular video*" OR "single video*" OR "ordinary video*" OR "single camera" OR "depth camera" OR smartphone* OR "smart phone*" OR "phone camera*" OR "camera phone*" OR "cell* phone*" OR video-based OR 2d OR 2-d OR "2 dimensional" OR "two dimensional" OR "pose estimation" OR openpose OR alphapose OR deepcut OR "deep learning" OR ai OR "machine learning") AND ("kinematic data" OR (track* N2 (body OR movement OR kinematic*)))) OR ((markerless OR marker-less OR "video recording*" OR "record* video*" OR "standard video*" OR "simple video*" OR "regular video*" OR "single video*" OR "ordinary video*" OR "single camera" OR "depth camera" OR "phone camera*" OR "camera phone*" OR video-based OR videotape* OR "video tap*" OR "pose estimation" OR openpose OR alphapose OR deepcut) AND ((assess* OR analy* OR evaluat* OR measur* OR parameter* OR impair* OR deviat* OR patholog* OR dysfunction*) N3 (movement OR motor OR motion OR gait OR balance OR postur* OR kinematic*))) OR "Leap motion" OR pose2sim OR kinect OR kinectv2 OR deeplabcut OR "Deep lab cut" OR trazer OR lidar OR "RGB-D camera" OR freemocap OR "Theia markerless" OR theia3d OR "Theia 3D" OR captury OR capturylive OR moveai OR "move AI" OR "iPi soft" OR "move.ai or simi shape 3d" OR "simi motion" OR ((vicon OR qualisys) AND markerless) OR "biomotion lab" OR "radical motion" OR opencap OR kinatrax

S2 ((fall OR falls OR falling) N3 (risk OR risks OR likelihood OR assess* OR evaluat* OR test OR determin* OR screen*)) OR frail* OR "functional evaluation*" OR "functional assessment*" OR "Functional analys*" OR "Fugl Meyer Assessment*" OR "stepping in place" OR "balance test*" OR "balance assessment*" OR "stepping test*" OR "timed up and go" OR "berg balance" OR "edmonton frail scale" OR "fried frailty" OR "frailty index" OR "comprehensive giereatric assessment" OR "clinical frailty scale" OR "pictorial fit-to-frail scale" OR "hierarchical assessment of balance and mobility" OR "IoRN" OR "SPARRA" OR "eFI" OR "FiND" OR "Easycare" OR "think frailty" OR "gérontopôle" OR "APOP" OR "ISAR" OR "PRISMA 7" OR "groningen frailty index" OR "frailty phenotype" OR "FRESH-screening" OR "TUGT" OR "frail scale" OR "falls screening" OR "MBESS" OR "BESTest" OR "Mini-BEST" OR "MD-UPDRS" OR mds-updrs OR "nine-hole peg" OR "9-hole peg" OR updrs OR uprds OR "box and block" OR "sit to stand" OR "one leg stance test" OR "forward functional reach" OR "functional reach test" OR "gait assessment" OR "functional gait assessment" OR "Tinetti gait assessment" OR "gait assessment and intervention tool" OR "dynamic gait index" OR "up and go test" OR "activities specific balance confidence scale" OR "gait and balance scale" OR "functional ambulation classification scale" OR ((parameter* OR impair* OR deviat* OR patholog* OR dysfunction*) N3 (movement OR motor OR motion OR gait OR balance OR postur* OR kinematic)) OR (gait N5 freez*)

(MH "Dementia+") OR (MH "Cardiovascular Diseases+") OR (MH "Parkinsonian Disorders+") OR (MH "Multiple Sclerosis+") OR (MH "Motor Neuron Diseases+") OR (MH "Amyotrophic Lateral Sclerosis") OR (MH "Muscular Dystrophy+") OR (MH "Renal Insufficiency, Chronic+") OR (MH "Liver Cirrhosis+") OR (MH "Brain Injuries+") OR (MH "Pulmonary Disease, Chronic Obstructive+") OR (MH "Neurodegenerative Diseases+") or dementia OR "heart failure" OR "heart disease" OR "cardiac disease*" OR "cardiovascular disease" OR hypertension OR stroke OR poststroke OR "transient ischemic attack*" OR "ischaemic attack*" OR "cerebrovascular accident*" OR alzheimer* OR parkinson* OR "multiple scleros*" OR "amyotrophic lateral sclerosis" OR "motor neuron disease" OR huntington* OR "muscular dystroph*" OR "chronic kidney diseas*" OR "chronic renal insufficiency" OR "chronic kidney injur*" OR "kidney failure" OR "end stage kidney disease" OR

cirrhosis OR "traumatic brain injur*" OR "acquired brain injur*" OR (chronic N2 ("lung disease" OR "pulmonary disease*")) OR copd OR "chronic obstructive pulmonary" OR ((degenerative OR neurodegenerative OR neurological) N4 (diseas* OR illness* OR disorder* OR condition*)) OR "over 65" OR "65 and over" OR "65 or over" OR "65+ years" OR frail* OR retired OR sarcopeni* OR elder* OR "old* old" OR "old age" OR centenarian* OR nonagenarian* OR octogenarian* OR septuagenarian* OR aging OR (senior* NOT (("high school" OR university OR college) N3 senior*)) OR gerontolog* OR geriatric* OR veteran* OR post-menopaus* OR postmenopaus* OR "mature adult*" OR "mature patient*" OR "mature individual*" OR "mature resident*" OR "aged adult*" OR "aged patient*" OR "aged individual*" OR "aged resident*" OR "aged donor*" OR "aged population*" OR "aged care" OR "nursing home resident*" OR "nursing home patient*" OR (older N2 (people OR person* OR client* OR resident* OR adult* OR patient* OR individual* OR donor* OR population* OR women OR men))

S4 (TI((animal-model* OR rat OR rats OR pig OR pigs OR porcine OR mouse OR mice OR murine OR hamster OR hamsters OR bovine OR sheep OR primate* OR monkey* OR zebra*) NOT human*)) OR (TI((child OR children OR infant* OR neonat* OR adolescen* OR teen* OR youths OR pediatric* OR paediatric*) NOT adult*)) OR (TI(exergam* OR "gamified exercis*" OR swim*))

S5 (S1 AND S2 AND S3) NOT S4 **Limiters** - English Language

Scopus (Advanced search)
Date searched: Oct 25, 2024
Results: 3382

(TITLE-ABS-KEY ("motion capture" OR mocap OR "motion tracking" OR "motion analysis" OR ((markerless OR marker-less) AND (video OR camera OR smartphone* OR "smart phone*" OR iphone* OR "cell* phone*")) OR ((markerless OR marker-less OR "video recording*" OR "record* video*" OR "video tap*" OR videotap* OR "standard video*" OR "simple video*" OR "regular video*" OR "single video*" OR "ordinary video*" OR "single camera" OR "depth camera" OR smartphone* OR "smart phone*" OR "phone camera*" OR "camera phone*" OR "cell* phone*" OR video-based OR 2d OR 2-d OR "2 dimensional" OR "two dimensional" OR "pose estimation" OR openpose OR alphapose OR deepcut OR "deep learning" OR ai OR "machine learning") AND ("kinematic data" OR (track* W/2 (body OR movement OR kinematic*))))) OR ((markerless OR marker-less OR "video recording*" OR "record* video*" OR "standard video*" OR "simple video*" OR "regular video*" OR "single video*" OR "ordinary video*" OR "single camera" OR "depth camera" OR "phone camera*" OR "camera phone*" OR video-based OR videotape* OR "video tap*" OR "pose estimation" OR openpose OR alphapose OR deepcut) AND ((assess* OR analy* OR evaluat* OR measur* OR parameter* OR impair* OR deviat* OR patholog* OR dysfunction*) W/3 (movement OR motor OR motion OR gait OR balance OR postur* OR kinematic*))) OR "Leap motion" OR pose2sim OR kinect OR kinectv2 OR deeplabcut OR "Deep lab cut" OR trazer OR lidar OR "RGB-D camera" OR freemocap OR "Theia markerless" OR theia3d OR "Theia 3D" OR captury OR capturylive OR moveai OR "move AI" OR "iPi soft" OR "move.ai or simi shape 3d" OR "simi motion" OR ((vicon OR qualisys) AND markerless) OR "biomotion lab" OR "radical motion" OR opencap OR kinatrax) AND TITLE-ABS-KEY (((fall OR falls OR falling) W/3 (risk OR risks OR likelihood OR assess* OR evaluat* OR test OR determin* OR screen*)) OR frail* OR "functional evaluation*"

OR "functional assessment*" OR "Functional analys*" OR "Fugl Meyer Assessment*" OR "stepping in place" OR "balance test*" OR "balance assessment*" OR "stepping test*" OR "timed up and go" OR "berg balance" OR "edmonton frail scale" OR "fried frailty" OR "frailty index" OR "comprehensive giereatric assessment" OR "clinical frailty scale" OR "pictorial fit-to-frail scale" OR "hierarchical assessment of balance and mobility" OR "IoRN" OR "SPARRA" OR "eFI" OR "FiND" OR "Easycare" OR "think frailty" OR "gérontopôle" OR "APOP" OR "ISAR" OR "PRISMA 7" OR "groningen frailty index" OR "frailty phenotype" OR "FRESH-screening" OR "TUGT" OR "frail scale" OR "falls screening" OR "MBESS" OR "BESTest" OR "Mini-BEST" OR "MD-UPDRS" OR mds-updrs OR "nine-hole peg" OR "9-hole peg" OR updrs OR uprds OR "box and block" OR "sit to stand" OR "one leg stance test" OR "forward functional reach" OR "functional reach test" OR "gait assessment" OR "functional gait assessment" OR "Tinetti gait assessment" OR "gait assessment and intervention tool" OR "dynamic gait index" OR "up and go test" OR "activities specific balance confidence scale" OR "gait and balance scale" OR "functional ambulation classification scale" OR ((parameter* OR impair* OR deviat* OR patholog* OR dysfunction*) W/3 (movement OR motor OR motion OR gait OR balance OR postur* OR kinematic)) OR (gait W/5 freez*)) AND (KEY (aged OR elderly OR "older adult*") OR TITLE-ABS-KEY (dementia OR "heart failure" OR "heart disease" OR "cardiac disease*" OR "cardiovascular disease" OR hypertension OR stroke OR poststroke OR "transient ischemic attack*" OR "ischaemic attack*" OR "cerebrovascular accident*" OR alzheimer* OR parkinson* OR "multiple scleros*" OR "amyotrophic lateral sclerosis" OR "motor neuron disease" OR huntington* OR "muscular dystroph*" OR "chronic kidney diseas*" OR "chronic renal insufficiency" OR "chronic kidney injur*" OR "kidney failure" OR "end stage kidney disease" OR cirrhosis OR "traumatic brain injur*" OR "acquired brain injur*" OR (chronic W/2 ("lung disease" OR "pulmonary disease*")) OR copd OR "chronic obstructive pulmonary" OR ((degenerative OR neurodegenerative OR neurological) W/4 (diseas* OR illness* OR disorder* OR condition*)) OR "over 65" OR "65 and over" OR "65 or over" OR "65+ years" OR frail* OR retired OR sarcopeni* OR elder* OR "old* old" OR "old age" OR centenarian* OR nonagenarian* OR octogenarian* OR septuagenarian* OR aging OR (senior* AND NOT (("high school" OR university OR college) W/3 senior*)) OR gerontolog* OR geriatric* OR veteran* OR post-menopaus* OR postmenopaus* OR "mature adult*" OR "mature patient*" OR "mature individual*" OR "mature resident*" OR "aged adult*" OR "aged patient*" OR "aged individual*" OR "aged resident*" OR "aged donor*" OR "aged population*" OR "aged care" OR "nursing home resident*" OR "nursing home patient*" OR (older W/2 (people OR person* OR client* OR resident* OR adult* OR patient* OR individual* OR donor* OR population* OR women OR men)))) AND NOT ((TITLE (animal-model* OR rat OR rats OR pig OR pigs OR porcine OR mouse OR mice OR murine OR hamster OR hamsters OR bovine OR sheep OR primate* OR monkey* OR zebra*) AND NOT TITLE (human*)) OR (KEY (animal-model* OR rat OR rats OR pig OR pigs OR porcine OR mouse OR mice OR murine OR hamster OR hamsters OR bovine OR sheep OR primate* OR monkey* OR zebra*) AND NOT KEY (human)) OR TITLE ((child OR children OR infant* OR neonat* OR adolescen* OR teen* OR youths OR pediatric* OR paediatric*) AND NOT adult*) OR TITLE (exergam* OR "gamified exercis*" OR swim*))) AND (LIMIT-TO (LANGUAGE , "English"))
